# Case report: A case of piriformis pyomyositis and pyogenic sacroiliitis due to non-typhoidal *Salmonella* bacteremia in an immunocompetent healthy adult

**DOI:** 10.3389/fmed.2024.1381555

**Published:** 2024-05-30

**Authors:** Atsuhiro Kanno, Kohei Suzuki, Daiki Narai, Akinobu Aihara, Takehito Ito, Takahiro Ohara, Kazuhiro Sumitomo, Katsutoshi Furukawa

**Affiliations:** ^1^Department of Community and General Medicine, Tohoku Medical and Pharmaceutical University, Wakabayashi Hospital, Sendai, Japan; ^2^Division of Geriatric and Community Medicine, Faculty of Medicine, Tohoku Medical and Pharmaceutical University, Sendai, Japan

**Keywords:** bacterial translocation, fat suppressed T2 weighted image, non-typhoidal *salmonella* bacteremia, piriformis pyomyositis, pyogenic sacroiliitis

## Abstract

Non-typhoidal *Salmonella* (NTS) rarely causes bacteremia and subsequent focal infections as an extraintestinal complication, even in immunocompetent adults. A 25-year-old man was hospitalized for several days with difficulty moving due to fever, acute buttock pain, and shivering. He had no recent or current respiratory symptoms and no clear gastrointestinal symptoms. Physical examination revealed mild redness around the left buttock and difficulty raising the left lower extremity due to pain, in addition to which blood tests showed high levels of inflammatory markers. His clinical course and laboratory findings suggested sepsis, and magnetic resonance imaging revealed a high-intensity area in the left piriformis muscle on diffusion-weighted imaging; therefore, acute piriformis pyomyositis was strongly suggested. Cephazolin was started upon hospitalization; however, blood and stool cultures proved positive for NTS, and the antibiotics were changed to ceftriaxone. Follow-up MRI showed a signal in the left piriformis muscle and newly developed left pyogenic sacroiliitis. On the 25th hospital day, a colonoscopy was performed to identify the portal of entry for bacteremia, which revealed a longitudinal ulcer in the sigmoid colon in the healing process. His buttock pain gradually improved, and the antibiotics were switched to oral levofloxacin, which enabled him to continue treatment in an outpatient setting. Finally, the patient completed seven weeks of antimicrobial therapy and returned to daily life without leaving any residual disability. Invasive NTS infection due to bacteremia is rare among immunocompetent adults. Piriformis pyomyositis and subsequent pyogenic sacroiliitis should be added to the differential diagnosis of acute febrile buttock pain. In the case of NTS bacteremia, the entry site must be identified for source control. Additionally, the background of the host, especially in such an immunocompetent case, needs to be clarified; therefore, the patient should be closely examined.

## Introduction

Non-typhoidal *Salmonella* (NTS) is a significant cause of diarrhea in immunocompetent adults in clinical practice. NTS mainly causes gastrointestinal (GI) symptoms and is related to mild or even asymptomatic enteritis, most of which follow a self-limiting course without antibiotics (1). However, there are rare cases of bacteremia caused by NTS infection, which can be associated with focal infections in various organs. *Salmonella* bacteremia and subsequent local infections have been reported primarily in immunocompromised patients (2). However, they have also been reported in immunocompetent patients without underlying disease (3–6). Some cases of invasive NTS infection may present with abscess formation requiring drainage (7–10). However, depending on the degree of local infection, symptoms may improve with antimicrobial therapy alone (11, 12).

We report a rare case of piriformis pyomyositis that progressed to sacroiliitis due to NTS bacteremia in an immunocompetent healthy adult, which was triggered by mild and latent enteritis and was successfully treated with antimicrobials without abscess drainage. Appropriate use of magnetic resonance imaging (MRI) enabled the early and timely diagnosis and assessment of therapeutic efficacy. Additionally, although it was unclear during the clinical course in this case, immunodeficiency sometimes likely exists among patients with NTS bacteremia. Therefore, the background of the host should be scrutinized as closely as possible.

## Case presentation

A 25-year-old Japanese male was transported to the emergency room for acute immobility due to left buttock pain for 2 days. The day before his transport, he visited a local orthopedic surgeon, where radiographs were used to evaluate his pain. However, there were no apparent abnormalities, and the patient was prescribed analgesics. Nevertheless, there was no improvement in the patient’s symptoms, and the patient had difficulty in daily living due to continued pain in his left buttock. Consequently, the patient was transported to the emergency department of our hospital. The patient had no remarkable medical history or underlying diseases and was not taking regular medication. The patient had no recent or current respiratory symptoms and no clear gastrointestinal symptoms. The patient was a nonsmoker and a social drinker. The patient had no recent travel history and had one dog and one cat.

**Figure 1:**
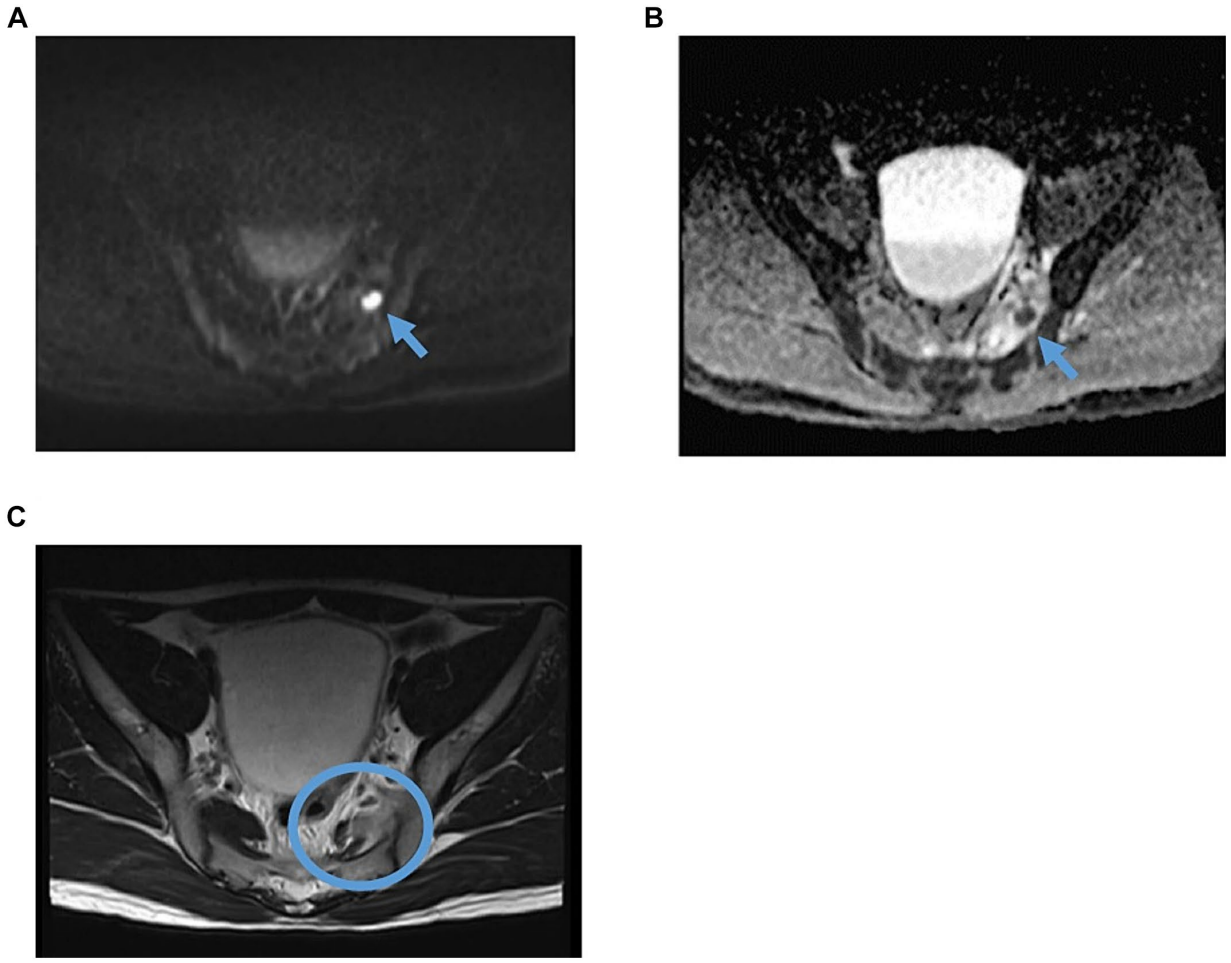
Pelvic MRI findings revealed piriformis pyomyositis on admission. **(A)** Axial DWI. The arrow indicates the nodal high-signal area within the piriformis muscle. **(B)** ADC. The arrow indicates a low-signal nodular area within the piriformis muscle is seen, a reasonable finding in the acute myositis. **(C)** Axial T2WI. The circle indicates a high-signal region with irregular edges in part of the left piriformis muscle, thought to be a finding of acute pyomyositis. DWI, diffusion-weighted imaging; ADC, apparent diffusion coefficient; T2WI, T2-weighted image.

**Figure 2:**
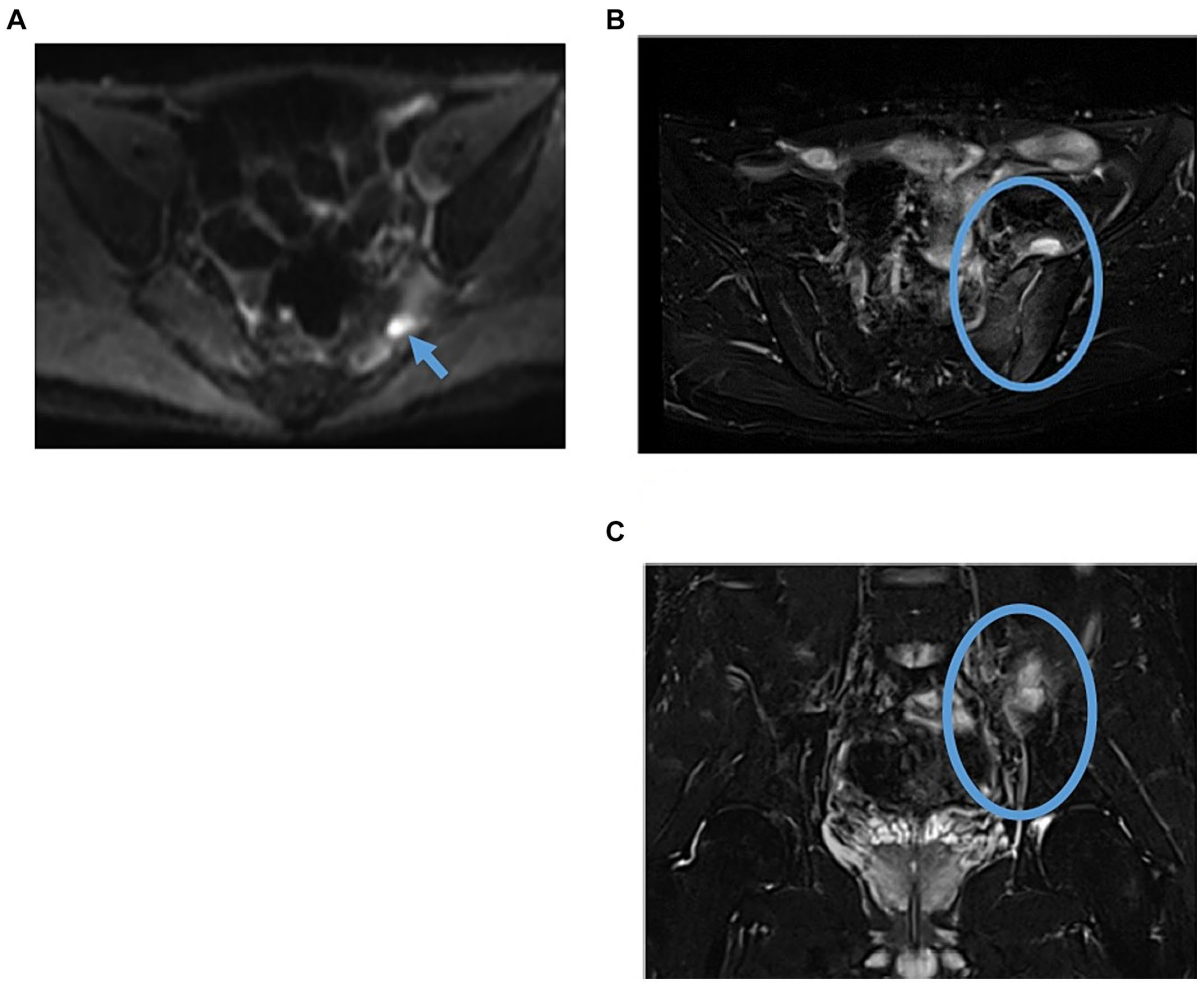
Follow-up pelvic MRI findings suggested piriformis pyomyositis and pyogenic sacroiliitis on the 9th hospital day. **(A)** Axial DWI. The arrow indicates an intra-articular nodular high-signal area in the piriformis muscle on the previous MRI, which tended to shrink. **(B)** Axial, fat-suppressed T2WI **(C)** Coronal fat-suppressed T2WI. The circle shows an elevated signal at the left sacroiliac joint site, suggesting pyogenic sacroiliitis. Additionally, fluid collection was noted on the ventral side. DWI, diffusion-weighted imaging; T2WI, T2-weighted image.

**Figure 3:**
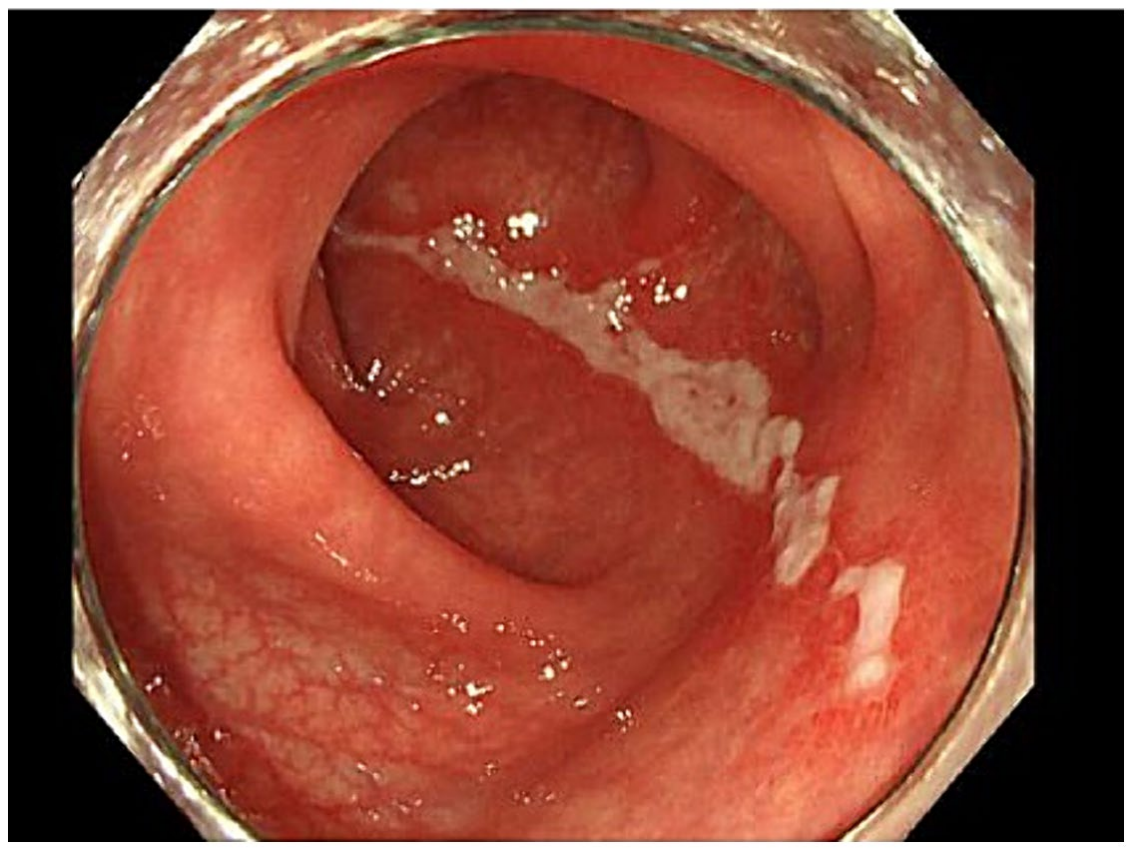
Longitudinal ulcer of the sigmoid colon. A 5 cm longitudinal ulcer lesion was found in the sigmoid colon on the anorectal side. The lesion was confined to the sigmoid colon.

In the emergency room, the patient’s vital signs were blood pressure of 107/63 mmHg, heart rate of 98 bpm with regular pulse, body temperature of 38.5°C, respiratory rate of 19 breaths/min, and blood oxygen saturation of 97% in room air. The patient was alert and oriented. Physical examination revealed tenderness of the left buttock with no palpable crepitus. Intense left gluteal and leg pain was evoked by passive extension and elevation of straight leg flexion with the knee. No heart murmur, abnormal lung sounds, or generalized lymphadenopathy was observed. He had no abdominal tenderness but showed left costovertebral angle (CVA) tenderness. Skin lesion was not also remarkable. Laboratory findings on admission were as follows: white blood count of 10,100 cells/μL, C-reactive protein (CRP) levels of 12.02 mg/dL, procalcitonin level of 0.68 ng/mL, sodium concentration of 137 mmol/L, and D-dimer level of 1.55 μg/dL (Table 1). Screening results were negative for human immunodeficiency virus (HIV) infection or diabetes as an underlying disease. The presence of left CVA tenderness recalled obstructive pyelonephritis due to a urinary stone, and an abdominal aortic dissection had to be ruled out as a differential diagnosis. Imaging studies showed no specific findings on contrast-enhanced computed tomography (CT); however, MRI showed a high signal on diffusion-weighted imaging (DWI) localized within the left piriformis muscle (Figure 1A), with a low-signal apparent diffusion coefficient (ADC) map in the same area (Figure 1B). In contrast, T2 weighted image (T2WI) showed increased signal and irregularity in part of the left piriformis muscle (Figure 1C), thought to be a finding of acute pyomyositis. The patient was referred to our department for further examination and treatment of the suspected piriformis pyomyositis. The patient was administered intravenous cephazolin 6 g daily. On day 3 after admission, a blood culture showed growth of *Salmonella* species. The antibiotic was switched to ceftriaxone 2 g intravenously daily. Blood cultures were performed a total of three times, including surveillance cultures; only the first blood culture was positive. Transthoracic echocardiography did not reveal vegetation and was negative for infective endocarditis. Additionally, stool culture results were positive for *Salmonella* species, non-pathogenic *Escherichia coli* and *Enterococcus* species. Spinal MRI was negative for pyogenic spondylitis, a metastatic infection. During this period, the patient experienced significant and persistent left buttock pain that was poorly managed. Due to pain, the patient was bedridden during hospitalization. A follow-up MRI of the pelvis was performed to reevaluate the disease status on the 9th day of the hospitalization. The high DWI high signal in the left piriformis muscle tended to shrink slightly (Figure 2A). The signal at the left sacroiliac joint was decreased on T1 weighted imaging (T1WI), and a high-signal area was revealed on a fat-suppressed T2WI. In addition, the iliac and sacral signals across the left sacroiliac joint increased on fat suppressed T2WI (Figures 2B,C), which suggested sacroiliac arthritis, and fluid collection was noted on the ventral side, suggestive of bursitis. A puncture of the lesion for culture and drainage was reserved for high-risk cases due to its small size, narrow lesion, and limited fluid collection. At this time, the patient’s spiking fever had improved, with only a low-grade fever in the 37°C range, and the patient was weaned off the bed with pain management and rehabilitation. On the 16th hospital day of hospitalization, the antibiotic was switched from ceftriaxone to oral levofloxacin 500 mg daily. Subsequently, *Salmonella* serotyping confirmed *Salmonella* Kentucky, which was detected in his blood and stool culture. Stool cultures were also examined from the patient’s pets, a dog and a cat; however, *Salmonella* spp. were not detected. GI endoscopy was performed to identify *Salmonella* bacteremia entry on the 25th hospital day. It could not be performed in the early days of hospitalization because the patient had significant buttock pain and could not be repositioned or held, which is essential for examination. Upper GI endoscopy revealed no abnormal findings; in contrast, lower GI endoscopy revealed a longitudinal ulcer covered with creamy white moss in the sigmoid colon (Figure 3), which were considered findings of the healing process of *Salmonella* enteritis. The condition of the ulcer just prior to its closure was consistent with the presumption that the onset of the disease was probably around the time of admission. The ulcer lesion was confined to the sigmoid colon, and the pathology was later revealed to be nonspecific inflammatory cell infiltrate consisting mainly of lymphocytes and there were no findings suggestive of malignancy. Based on these results, the patient was diagnosed with NTS bacteremia associated with bacterial translocation from sigmoid colon ulcers caused by NTS enteritis, leading to pyogenic myositis of the piriformis muscle and sacroiliitis. Pre-discharge MRI showed resolution of the piriformis muscle lesion and reduced ventral fluid retention; however, the fat-suppressed T2WI high-signal area around the left sacroiliac joint became more defined. Rather than focusing on the worsening MRI findings, our team focused on improving the clinical symptoms; it was considered that the patient was in a condition to be discharged from the hospital. The patient completed a seven week course of antimicrobial therapy, including the post-discharge outpatient clinic, until the patient was relieved of symptoms and the patient’s erythrocyte sedimentation rate and CRP levels were normalized. At the outpatient visit, the patient’s pyogenic sacroiliitis had improved on follow-up pelvic MRI. He was instructed to return for subsequent outpatient visits in case of another fever or gastrointestinal symptoms, and he returned to daily life without any residual disability.

**Table 1:** Laboratory findings on admission.

	Values	Normal range		Values	Normal range
Complete blood count			Blood chemistry		
WBC (×10^3^/μL)	10.1	3.3–8.6	Total protein (g/dL)	6.7	6,6–8.1
Neutrophil (%)	93.0		Albumin (g/dL)	4.1	4.1–5.1
Lymphocyte (%)	3.0		T-Bil (mg/dL)	1.71	0.4–1.5
Eosinophile (%)	0.0		AST (U/L)	19	13–30
RBC (×10^6^/μL)	4.63	3.86–4.92	ALT (U/L)	15	7–23
Hemoglobin (g/dL)	14.7	11.6–14.8	LDH (U/L)	180	124–222
Hematocrit (%)	43.2	35.1–44.4	ALP (U/L)	54	38–113
MCV (fL)	93.5	83.6–98.2	GGT (U/L)	16	9–32
Platelet (×10^3^/μL)	166	158–348	CPK (U/L)	135	41–153
Coagulation			BUN (mg/dL)	12	8–20
PT-INR	1.2	0.85–1.15	Creatinine (mg/dL)	0.92	0.46–0.79
FDP (μg/mL)	3.8	≤4.0	eGFR (mL/min/1.73m^2^)	84	60≤
D-dimer (μg/mL)	1.55	≤1.0	Na (mmol/L)	137	138–145
Fibrinogen (mg/dL)	512	150–400	K (mmol/L)	3.6	3.6–4.8
APTT (sec)	36.6	24.3–36.0	CRP (mg/dL)	12.02	0–0.14
BGA			PCT (ng/mL)	0.68	
pH	7.413	7.35–7.45	HbA1c (%)	5.4	4.9–6.2
pCO_2_ (mmHg)	40.5	35.0–45.0	Immunology		
pO_2_ (mmHg)	63.8	80≤	IgG (mg/dL)	931	870–1700
HCO_3_^−^ (mmol/L)	25.4	20–26	Ferritin (ng/dL)	975	39.4–340
Lactate (mmol/L)	2.3	0.5–1.5	IGRA	–	
Urinalysis			RPR	–	
pH	6.0	5.0–8.0	TPHA	–	
Occult blood	–		HBs-Ag	–	
Protein	–		HCV-Ab	–	
Leukocyte esterase	–		HIV Ag/Ab	–	

WBC, white blood cell count; RBC, red blood cell count; MCV, mean corpuscular volume; PT-INR, prothrombin time-international normalized ratio; FDP, fibrinogen/fibrin degradation products; APTT, activated partial thromboplastin time; BGA, blood gas analysis; AST, aspartate transaminase; ALT, alanine transaminase; LDH, lactate dehydrogenase; ALP, alkaline phosphatase; GGT, gamma-glutamyl transferase; CPK, creatine phosphokinase; BUN, blood urea nitrogen; eGFR, estimated glomerular filtration rate; CRP, c-reactive protein; PCT, procalcitonin; HbA1c, hemoglobin A1c; IGRA, interferon-gamma release assay; RPR, rapid plasma regain; TPHA, treponema pallidum particle agglutination; HBs-Ag, hepatitis B surface antigen; HCV-Ab, hepatitis C virus antibody; HIV Ag/Ab, human immunodeficiency virus antigen/antibody.

## Discussion

Here, we present a case of pyomyositis of the piriformis muscle complicated with sacroiliitis due to primary NTS bacteremia, which developed as acute buttock pain in an immunocompetent healthy adult. Pyomyositis is rare; and it is extremely rare for pyomyositis due to NTS bacteremia to occur in an immunocompetent individual, as in this case. Additionally, it is also an uncommon case that pyogenic enterocolitis progressed to a subsequent sacroiliitis and was treated with conservative treatment with antimicrobial therapy without requiring surgical drainage and successful without sequelae. Among several reports of pyomyositis and sacroiliitis caused by primary NTS bacteremia, most previous cases had unknown etiology (12, 13). Notably, mild and latent NTS enteritis was considered the cause of bacteremia, and the entry portal of bacteremia was identified as the sigmoid colon ulcer, which was the most intriguing aspect of our case.

NTS usually results in a self-limiting GI illness; however, it can also cause bacteremia and other focal infections. Invasive NTS are typically not associated with abdominal symptoms (14). However, it presents as a non-specific febrile illness, which is clinically difficult to differentiate from other infectious diseases. It is complicated with a higher case fatality rate than that seen with non-invasive infections (15, 16). *Salmonella* infections are usually caused by ingestion of contaminated food and water. It can also be acquired via the fecal-oral route from humans, farm animals or pets. It has been reported that NTS causes bacteremia in 5–15% of patients with GI symptoms (15, 17). Host factors that increase susceptibility to NTS invasive diseases, such as bacteremia, meningitis, or focal infection, include age (infants, young children, and older individuals), immunity (immunocompromised or malnourished individuals), and health status (recent malaria or HIV infection) (18, 19), none of which were applicable in our patient. However, in cases of NTS bacteremia with mild or no GI symptoms, it is supposed that two hypotheses could exist in the pathogenesis of developing NTS bacteremia. First, possible causes of immunocompromised conditions, such as malignancy, HIV infection, and hematological and rheumatologic disease, did not apply to the present case. However, another latent immunocompromised background may prevent a normal immune response from eliciting NTS, resulting in mild gastroenteritis symptoms even when the ulcerative lesions were formed in the sigmoid colon. Previously, it was reported that, in such patients, mucosal immunity may be compromised to the extent that the infective dose required for bacteremia is less than for severe gastroenteritis (20). The development of *Salmonella* bacteremia in the absence of recent or current gastroenteritis should prompt clinicians to evaluate an immunosuppressive condition. Second, there is a possibility that, due to underlying immunocompromising factors which was not detected in the present case, NTS might remain dormant in the reticuloendothelial system following a previous episode of gastroenteritis and re-emerge years later when cell-mediated immunity is impaired (21). These could also explain the development of NTS bacteremia with mild or no GI symptoms. There is a report on underlying immunological conditions that compromise Th1 immune responses and are related to an increased risk of *Salmonella* bacteremia (14); however, no detailed immune evaluation was performed in the present case.

As for the route of transmission, fecal cultures of the patient’s pets, a dog, and a cat, were performed, however, *Salmonella* was not detected. In addition, the patient had no daily contact with reptiles. He processed food, packed fresh vegetables, and never handled poultry. In Japan, the Food Sanitation Law mandates regular stool culture tests for food-related workers; this patient was also tested regularly, and no specific bacteria had yet been detected. However, these results about the patient were not necessarily deniable of a chronic asymptomatic carrier of NTS and the possibility of bacterial translocation leading to NTS bacteremia based on chronic intestinal NTS infection.

As the present treatment strategy at the timing of the absence of an identified organism on primary surveys in the emergency department suspected primary pyomyositis, we considered the most common cause of primary pyomyositis to be *Staphylococcus aureus*, and the patient was immunocompetent and healthy; therefore, we selected cefazolin as initial inpatient treatment. However, due to a blood culture test, NTS was reported to be detected. Based on the frequency of the trend of extraintestinal infection, preferred antimicrobial agents for treating nontyphoidal *Salmonella* bacteremia include a third-generation cephalosporin or fluoroquinolone, given these agents’ high tissue and intracellular concentrations. During inpatient management, treatment was continued with intravenous ceftriaxone. When discharge was possible, antibiotics were switched to levofloxacin, which has a high bioavailability, and he was followed up as an outpatient.

Conversely, the risk factors for pyomyositis in the piriformis muscle are young age, exercise or muscle trauma, immunocompromised status (diabetes mellitus, injection drug use, rheumatologic conditions, malnutrition, cirrhosis, and renal disease), and recent bloodstream infections as in the present case. These are, in part, common risk factors for NTS bacteremia. The most common pathogens in pyomyositis are *Staphylococcus aureus* and methicillin-resistant *Staphylococcus aureus* (22). *Salmonella* spp. are unlikely to be the causative pathogens in immunocompetent healthy adults. In this case, pyomyositis was associated with bacteremia and can be considered primary pyomyositis, in contrast to secondary pyomyositis, which contiguously spreads to the muscle from an adjacent infection. Pyomyositis is categorized into three stages representing disease progression from diffuse inflammation to focal abscess to sepsis (23). The present case belonged to stage 2, with skin findings and systemic symptoms suggestive of infection, a highly elevated white blood cell counts on examination, and pus accumulation depending on the condition.

In this case of pyomyositis and pyogenic sacroiliitis, MRI without gadolinium was reported to be useful in the diagnosis and follow-up to determine the efficacy of treatment (24–26) because MRI is highly sensitive to abscesses, muscle inflammation, and infection of adjacent structures when a lesion is suspected that cannot be identified even by contrast-enhanced computed tomography scan, such as a soft tissue infection. MRI has been proven to play an important role in the early diagnosis of pyomyositis; therefore, prompt, and correct diagnosis at an early stage prevents conditions requiring surgical invasion or further deterioration of the general condition, resulting in a cure following treatment with antimicrobial agents. Additionally, regarding MRI changes over time, which are related to sacroiliitis, it has been reported that there is a dissociation between early clinical improvement and MRI findings and that MRI signal changes may persist for several months (27). Our team followed this principle when deciding to discharge the patient from the hospital. Clinical symptoms and non-imaging laboratory findings were used as the primary references.

Our case report has limitations. First, the possibility that the sigmoid colon ulcer was an active lesion at the onset was not verified by real-time colonoscopy due to the patient’s status. Second, we carefully excluded the common causes of immunodeficiency; a more detailed examination was not performed. Third, the causative organism for a piriformis abscess was not definitively clarified because the degree of pus accumulation was minimal, and puncture into the piriformis abscess was withheld.

In conclusion, we presented a case of piriformis pyomyositis and pyogenic sacroiliitis caused by NTS bacteremia due to mild or latent enteritis that represented acute buttock pain in an immunocompetent adult. In such a case, it is necessary to examine the entry site of bacteremia as thoroughly as possible. Furthermore, this is the robust approach to life-saving and curative treatment. Early MRI evaluation allowed for prompt diagnosis and appropriate assessment of therapeutic efficacy. Rapid, definitive diagnosis and adequate antimicrobial treatment enabled the patient to return to society without sequelae. Piriformis pyomyositis and pyogenic sacroiliitis should be considered differential diagnoses for acute febrile buttock pain, even in immunocompetent patients. In cases of NTS bacteremia, patient background related to immunodeficiency should be fully sought, in addition to detecting the portal entry.

## Data availability statement

The original contributions presented in the study are included in the article, further inquiries can be directed to the corresponding author.

## Ethics statement

Written informed consent was obtained from the individual for the publication of any potentially identifiable images or data included in this article.

## Author contributions

AK: Conceptualization, Data curation, Formal analysis, Investigation, Methodology, Project administration, Supervision, Validation, Visualization, Writing – original draft, Writing – review & editing. KoS: Data curation, Investigation, Writing – original draft. DN: Data curation, Investigation, Writing – original draft. AA: Investigation, Writing – original draft. TI: Conceptualization, Investigation, Writing – review & editing. TO: Investigation, Methodology, Writing – review & editing. KaS: Data curation, Investigation, Methodology, Writing – review & editing. KF: Conceptualization, Project administration, Supervision, Visualization, Writing – original draft, Writing – review & editing.
